# Jointly Learning Non-Cartesian *k*-Space Trajectories and Reconstruction Networks for 2D and 3D MR Imaging through Projection

**DOI:** 10.3390/bioengineering10020158

**Published:** 2023-01-24

**Authors:** Chaithya Giliyar Radhakrishna, Philippe Ciuciu

**Affiliations:** 1 Neurospin, Commissariat à L’énergie Atomique et Aux Énergies Alternatives (CEA), Centre National de la Recherche Scientifique (CNRS), Université Paris-Saclay, 91191 Gif-sur-Yvette, France; 2 Inria, Models and Inference for Neuroimaging Data (MIND), 91120 Palaiseau, France

**Keywords:** MRI, non-Cartesian, *k*-space trajectories, reconstruction networks

## Abstract

Compressed sensing in magnetic resonance imaging essentially involves the optimization of (1) the sampling pattern in *k*-space under MR hardware constraints and (2) image reconstruction from undersampled *k*-space data. Recently, deep learning methods have allowed the community to address both problems simultaneously, especially in the non-Cartesian acquisition setting. This work aims to contribute to this field by tackling some major concerns in existing approaches. Particularly, current state-of-the-art learning methods seek hardware compliant *k*-space sampling trajectories by enforcing the hardware constraints through additional penalty terms in the training loss. Through ablation studies, we rather show the benefit of using a projection step to enforce these constraints and demonstrate that the resulting *k*-space trajectories are more flexible under a projection-based scheme, which results in superior performance in reconstructed image quality. In 2D studies, our novel PROjection for Jointly lEarning non-Cartesian Trajectories while Optimizing Reconstructor (PROJeCTOR) trajectories present an improved image reconstruction quality at a 20-fold acceleration factor on the fastMRI data set with SSIM scores of nearly 0.92–0.95 in our retrospective studies as compared to the corresponding Cartesian reference and also see a 3–4 dB gain in PSNR as compared to earlier state-of-the-art methods. Finally, we extend the algorithm to 3D and by comparing optimization as learning-based projection schemes, we show that data-driven joint learning-based PROJeCTOR trajectories outperform model-based methods such as SPARKLING through a 2 dB gain in PSNR and 0.02 gain in SSIM.

## 1. Introduction

A major challenge limiting the use of Magnetic Resonance Imaging (MRI) is long acquisition times, arising due to short decay of the MR signal, which is used to sample multi-dimensional *k*-space data through numerous and repetitive radio-frequency pulses. Using Compressed Sensing (CS) theories [1], significant speed up can be obtained by undersampling the *k*-space according to Variable Density Sampling (VDS) [2,3,4,5,6], whose shape depends on the underlying anatomy, contrast and coil structure. Non-Cartesian sampling can be used to efficiently achieve VDS of *k*-space, as this type of sampling, which relies on curves, is more flexible and efficient compared to straight lines used in traditional Cartesian acquisitions. While conventional non-Cartesian sampling patterns such as spiral, radial, rosette, etc. [7,8,9,10,11,12], have been proposed in the literature and can sample the *k*-space according to VDS, they do not sample at a well-defined user specified Target Sampling Density (TSD). Tailoring such non-Cartesian trajectories according to a MR imaging protocol and a given TSD is hard, as these *k*-space sampling curves or trajectories are constrained by the MR hardware limits, notably on the maximum gradient magnitude $G_{\max}$ and slew rate $S_{\max}$.

To meet these constraints in a safe manner, the Spreading Projection Algorithm for Rapid K-space sampLING (SPARKLING) was introduced in [13,14], and then extended to 3D [15] as an iterative procedure to optimize a non-Cartesian *k*-space sampling pattern according to a prescribed TSD. Such patterns are typically segmented into multiple shots or *k*-space trajectories, each of them being compliant with the above-mentioned MR hardware constraints. Further, the algorithm results in locally uniform sampling patterns, and thus avoids holes and clusters in *k*-space. However, the SPARKLING is a model-driven framework, which is characterized by a TSD that needs to be known in advance to feed the optimization process. Previously, in [16], to address this issue, we learned the TSD in a deep learning setting using LOUPE [17] as an acquisition model. Although this allowed us to improve the reconstruction performances, there was still a mismatch in the learning process. Using LOUPE [17], gridded TSD was learned in the Cartesian domain, while the actual trajectory being optimized was non-Cartesian. Additionally, we had to learn a different non-Cartesian image reconstruction model (e.g., a convolutional neural network or CNN) that was disconnected from the optimized trajectories, making the overall process computationally expensive. Further, as such disjointedness between training a TSD and testing on different non-Cartesian trajectories and image reconstruction neural nets could lead to suboptimal results, there is a need to jointly learn both the TSD and the image reconstruction deep learning architecture in a non-Cartesian setting.

Recently, new methods [18,19,20] have been developed to overcome the need for estimating a TSD, through direct joint learning of the non-Cartesian *k*-space sampling trajectories and MR image reconstruction in a data-driven manner on the fastMRI dataset [21]. In [18,20], the authors jointly learned Physics-informed learned optimal trajectories (PILOT) trajectories along with U-net parameters as a reconstruction model to denoise the basic image yielded by the adjoint of the Non-Uniform Fast Fourier Transform (NUFFT) operator. However, this method relies on auto-differentiation of the NUFFT operator, which is inaccurate numerically as observed in [22], resulting in sub-optimal local minima. This suboptimality was actually reflected in the final shape of the learned trajectories, which only slightly deviated from their initialization.

In B-spline parameterized Joint Optimization of Reconstruction and K-space trajectories (BJORK) [19], the authors use [22] to obtain a more accurate Jacobian approximation of the NUFFT operator. Both above-referenced approaches [18,19] enforced the hardware constraints by adding penalty terms to the the loss that is minimized during training. Although a viable option, this requires tuning a hyper-parameter associated with each of these penalty terms in the cost function. Moreover, it does not guarantee that the optimized trajectories will strictly meet these constraints. Further, these penalty terms affect the overall gradients of the loss function, thereby resulting in suboptimality of the trajectories. In BJORK [19], the trajectories were parameterized with B-spline curves in order to reduce the number of trainable parameters. Although this strategy drastically minimizes the search space and the training time, such parameterization severely limits the degrees of freedom of the trajectories and prevents them from an improved exploration of the *k*-space. Finally, both methods do not make use of Density Compensators (DCp), which plays a key role in obtaining clearer MR images in the non-Cartesian deep learning setting [23].

In this work, we first develop a generic model for PROJeCTOR. More precisely, we introduce a method that learns the *k*-space trajectories in a data-driven manner while embedding a projected gradient descent algorithm [24] to fulfill the hardware constraints during the training stage. Unlike BJORK, we directly learn the *k*-space sampling trajectories and use multi-resolution [25] similar to the SPARKLING to limit the number of trainable parameters at each step. Then, we compare these PROJeCTOR results to two state-of-the-art methods, PILOT [18] and BJORK [19] in 2D MRI. In a more controlled setting, we show the importance of the projection step during the optimization of *k*-space trajectories and demonstrate its superiority over penalty-based methods like PILOT and BJORK to enforce hardware constraints. Finally, we compare and show the superiority of data-driven PROJeCTOR trajectories compared to model-based non-Cartesian SPARKLING trajectories.

## 2. Materials and Methods

**Figure 1:**
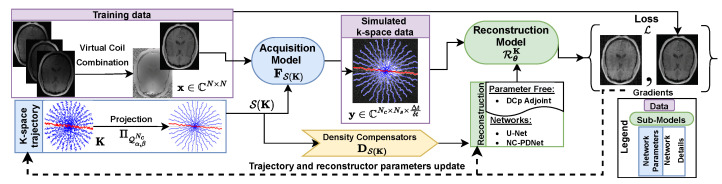
A generic learning-based framework for joint optimization of the MRI acquisition and reconstruction models. This framework consists of two sub-models: (1) the acquisition model $F_{S(K)}$ parameterized by the k-space sampling trajectories **K** and interpolated through linear interpolation S(K), and (2) the reconstruction model $R_{\theta }^{K}$ parameterized by θ. The input training data consists of emulated single coil complex images, from which simulated k-space data are obtained through $F_{S(K)}$. The loss L is calculated between the reconstructed image and the ground truth. The gradients are backpropagated to result in a k-space trajectory and reconstructor parameters update. Projection $\Pi_{Q_{\alpha ,\beta }^{N_{c}}}$ is carried out after the trajectory update to make sure it satisfies the hardware constraints and lies in the constraint set $Q_{\alpha ,\beta }^{N_{c}}$. Further, the density compensator $D_{S(K)}$ of the k-space trajectory serves as input to the reconstruction network.

In this section, we present a generic and modular framework (Figure 1) for learning non-Cartesian *k*-space trajectories and deep neural networks for MR image reconstruction. Particularly, we discuss 2 sub-models; namely, (1) an acquisition model parameterized by *k*-space trajectory, and (2) a reconstruction model parameterized by a deep neural network. Later, we discuss in detail how to handle the MR hardware constraints and which approach seems the most efficient within the sampling pattern optimization process to end up with hardware compliant *k*-space trajectories.

### 2.1. Data and Preprocessing

In order to reduce the memory footprint and the training time, we did not process multicoil *k*-space data as input in the pipeline shown in Figure 1. Instead, we learn the trajectories and image reconstruction model on emulated single coil data obtained using a virtual coil combination [26] of per-channel images. This is done through phase reconstruction from multi-coil data through the use of a virtual reference coil. This virtual-reference coil is generated as a weighted combination of measurements from all receiver coils. The multiple phase-corrected coil complex images are combined using the inverse covariance matrix, to result in a complex image with optimal estimates of the absolute magnetization phase (see [26] for mathematical details).

Overall, we rely on notations developed in [15], and we assume isotropic resolution and FOV with matrix size in each axis as *N*. This assumption is purely for notational convenience and does not limit the applicability of our framework to isotropic data. If *D* is the imaging dimension, we denote a MR image or volume as $x\in C^{N^{D}}$, over a field of view $F^{D}$. Throughout the manuscript, we refer to x as MR image, while it can be MR volume when D=3. The *k*-space of this acquisition is defined in ${\left[-K_{\max},K_{\max}\right]}^{D}$, with $K_{\max}=\frac{N}{2F}$. However, for the sake of simplicity, we normalize the *k*-space to $\Omega ={\left[-1,1\right]}^{D}$. For both 2D and 3D imaging, we take observation time (*T*_Obs_) = 5.12 ms (readout time), giving us $N_{s}=512$ samples per trajectory (see details in Section 2.2). This readout value is fully compatible with those used in T1- and T2-weighted imaging.

For our experiments in 2D imaging, we used the fastMRI brain MR data set [21], which consists of 1447 $T_{1}$ and 2678 $T_{2}$-weighted images with N=320. In contrast, for validation in 3D imaging, we used the Calgary brain data set [27], which consists of 167 $T_{1}$-w MR volumes at 1 mm isotropic sagittal acquisitions, with matrix size 256×224×170. For both imaging protocols, we used an Acceleration Factor (AF) = $\frac{N^{D-1}}{N_{c}}$ of 20 (see [15]), resulting in a number of trajectories $N_{c}=16$ for 2D imaging and $N_{c}=1681$ for 3D imaging (see details in Section 2.2).

### 2.2. K-Space Trajectory (***K***)

The acquisition model is parameterized by a *k*-space sampling pattern K, which is composed of $N_{c}$ shots, $K={\left(k_{i}\right)}_{i=1}^{N_{c}}$. Each shot can be played by the scanner hardware at the pace of gradient raster time Δt, throughout the readout time $T_{obs}$, resulting in $N_{s}=\left\lfloor \frac{T_{\mathrm{obs}}}{\Delta t}\right\rfloor$ samples per shot and an overall sampling pattern as $K\in \Omega^{N_{c}\times N_{s}}$.

The *k*-space trajectories are constrained in speed and acceleration by the maximum gradient strength $G_{\max}$ and maximum slew rate $S_{\max}$, respectively. Additionally, affine constraints are added to the trajectory design to ensure that the center of *k*-space is sampled at Echo Time (TE) in every shot, resulting in a stable and required target contrast of reconstructed MR images. From [15,24], we model these constraints as follows:

(1) $$Q_{\alpha ,\beta }^{N_{c}}=\left\{\begin{array}{c} \forall i=1,\ldots ,N_{c},\;k_{i}\in R^{3\times N_{s}},\\ Ak_{i}=v,\\ {∥k_{i}∥}_{\infty }\leq 1,\;∥{\dot{k}}_{i}{∥}_{2,\infty }\leq \alpha ,\;{∥{\ddot{k}}_{i}∥}_{2,\infty }\leq \beta , \end{array}\right\}$$

where

$$\begin{array}{cc} {\dot{k}}_{i}\left[n\right] & =\frac{k_{i}\left[n\right]-k_{i}\left[n-1\right]}{\Delta t}\\ {\ddot{k}}_{i}\left[n\right] & =\frac{k_{i}\left[n+1\right]-2k_{i}\left[n\right]+k_{i}\left[n-1\right]}{\Delta t^{2}}\\ {∥c∥}_{2,\infty } & =\underset{0\leq n\leq N_{s}-1}{\sup}{\left(|c_{x}{\left[n\right]|}^{2}+|c_{y}{\left[n\right]|}^{2}+{\left|c_{z}\left[n\right]\right|}^{2}\right)}^{1/2}, \end{array}$$

for all $c\in \Omega^{N_{s}}$, and (α,β) are obtained by normalizing hardware and Nyquist constraints to the sampling domain Ω (from [13]):

(2a) $$\begin{array}{cc} \alpha & =\frac{1}{K_{\max}}\min\left(\frac{\gamma G_{\max}}{2\pi },\frac{1}{FOV\cdot \delta t}\right) \end{array}$$

(2b) $$\begin{array}{cc} \beta & =\frac{\gamma S_{\max}}{2\pi K_{\max}} \end{array}$$

The TE point constraints are modeled through **A** and **v** in (1) (see [24] for details and more complex affine constraints). A and v are tailored to have the following equivalent expression on each *k*-space trajectory:

(3) $$k_{i}^{d}\left[k_{\mathrm{te}}\right]=0\;\;\begin{array}{c} \forall \;i\in \{1,\ldots N_{c}\},\\ \forall \;d\in \{x,y,z\},\\ k_{\mathrm{te}}=\left\lfloor \frac{TE}{\Delta t}\right\rfloor \;. \end{array}$$

### 2.3. Acquisition Model ($F_{k}$)

With the *k*-space sampling pattern K, we model the acquisition process at the MR scanner with non-uniform fast Fourier transform (NUFFT) [28] operator **F_K_**. However, in practice, the *k*-space data is sampled in analog-to-digital converter (ADC) at every dwell time δt, with $o=\frac{\Delta t}{\delta t}\geq 1$ the oversampling factor along each trajectory. Thus, a more realistic acquisition model of *k*-space data $y\in C^{N_{c}\times N_{s}\times o}$ is:

(4) $$\begin{array}{cc} y & =F_{S(K)}x+\epsilon \end{array}$$

where S is the linear interpolator, which interpolates the *k*-space trajectory to have $o\times N_{s}$ samples during readout, to model the oversampling by ADC, and ϵ is the simulated noise, which is already present in the data set as it is prospectively acquired by the MR system.

As the *k*-space trajectories are non-Cartesian, this creates a variable density sampling in *k*-space, due to which a simple adjoint of NUFFT operator $F_{K}^{*}$ is not close to the inverse operator and is not sufficiently accurate to reconstruct a clear MR image. To prevent this, a density compensation (DCp) mechanism has been introduced in the non-Cartesian image reconstruction community for more than 20 years [29]. It allows us to more fairly balance the weights of *k*-space samples associated with the low and high frequencies during the iterative reconstruction process. Following this principle, we obtained **D**_*S*(**K**)_ for the linearly interpolated *k*-space trajectory S(K), which is computed by 10 iterations of the algorithm described in [29]. As noted in [23], DCp is crucial for deep learning-based reconstruction to avoid numerical issues and result in better reconstructed image quality.

### 2.4. Reconstruction Model: Deep Neural Network ($R_{K}^{\theta }$)

The reconstruction network $R_{K}^{\theta }$ is a deep neural network that reconstructs an MR image $\hat{x}$ from the *k*-space data y and the *k*-space trajectory S(K). The estimated DCp are also provided as input to the network, to better condition the reconstruction problem, resulting in faster convergence and giving us:

(5) $$\begin{array}{c} \hat{x}=R_{K}^{\theta }\left(y,D_{S(K)}\right)\;. \end{array}$$

A simple parameter-free reconstruction would be the density compensated adjoint, i.e., $R_{K}^{\theta }=D_{S(K)}F_{K}^{*}$. To go further, we implemented the density compensated non-Cartesian primal dual network (NC-PDNet [23]) as the reconstruction network. The latter alternates between a data consistency step in *k*-space and convolutional neural network (CNN) based denoising in the image domain with kernel size 3×3 in 2D and 3×3×3 in 3D. We used the same network architecture as in [23] except that this time we expanded the architecture over 12 unrolled iterations, and the number of filters per iteration $N_{f}$ = 32 filters.

### 2.5. Loss, Gradients and Optimizer

The reconstruction error used as loss function $L_{r}$ (between the reference MR image **x** and its reconstruction $\hat{x}$) in this study was inspired by [30] and is defined as a weighted sum of $\ell_{1}$, $\ell_{2}$ and the multi-scale structural similarity index (*S*) [31]:

$$\begin{array}{cc} L_{r}\left(x,\hat{x}\right) & =\alpha \left(1-S\left(x,\hat{x}\right)\right)+\bar{\alpha }||x-\hat{x}{\left|\right|}_{1}+\frac{{\bar{\alpha }}^{2}}{2}||x-\hat{x}{\left|\right|}_{2} \end{array}$$

with $\bar{\alpha }=1-\alpha$, and the value of α was tuned to 0.995 to give nearly equally balanced loss terms. The training was carried out by minimizing reconstruction loss $L_{r}$ with respect to both parameters θ of the reconstruction network and *k*-space trajectory **K** as follows:

(6) $$\begin{array}{cc} \left(\hat{K},\hat{\theta }\right) & =\underset{\left(K\in Q_{\alpha ,\beta }^{N_{c}},\theta \right)}{\arg\;\min}L_{r}\left(x,R_{K}^{\theta }\left(FS_{\left(K\right)}x\right)\right) \end{array}$$

For optimizing the trajectory **K**, we derived the gradient of the loss function $L_{r}$ with respect to **K**:

(7) $$\begin{array}{cc} \frac{\partial L_{r}\left(x,\hat{x}\right)}{\partial K} & =\nabla L_{r}\left(x,\hat{x}\right)\frac{\partial \hat{x}}{\partial K}=\nabla L_{r}\left(x,\hat{x}\right)\frac{\partial R_{K}^{\theta }\left(y\right)}{\partial K} \end{array}$$

For ease of mathematical derivation, here we take the case of a parameter-free reconstruction as described in Section 2.4 with $R_{K}^{\theta }=D_{S(K)}F_{S}^{*}(K)$. In order to simplify this gradient calculation and reduce its computational complexity, we neglect the contribution of gradients from density compensators $D_{S(K)}$. This contribution of gradients from $D_{S(K)}$ was also ignored in realistic implementations to reduce gradient computation time and GPU memory requirements. These assumptions lead to the following gradient expression:

$$\begin{array}{cc} \frac{\partial L_{r}}{\partial K} & =\nabla L_{r}\left(\frac{\partial \hat{x}}{\partial D_{S(K)}y}D_{S(K)}\frac{\partial \left(F_{S(K)}x\right)}{\partial K}+\frac{\partial F_{S(K)}^{*}}{\partial K}\right) \end{array}$$

In order to compute the gradient of NUFFT operators $F_{S(K)}$ and $F_{S(K)}^{*}$ with respect to the *k*-space trajectory **K**, we used [22] to obtain a fast and accurate approximation of the Jacobians. As these underlying gradients vary extremely in norm depending on the *k*-space region (as noted in [32]), we used the ADAM optimizer for learning the trajectories, while we relied on a rectified-ADAM solver for optimizing the image reconstruction network $R_{\theta }^{K}$.

During training, the gradient descent was carried out stochastically with a batch size of 64 in 2D, while due to memory limitations, it was limited to 1 in 3D. However, as the gradients with respect to *k*-space trajectory were extremely noisy for this low batch size in 3D, we relied on a smaller learning rate of $2\times 10^{-4}$ as compared to $10^{-3}$ in 2D runs. On the other hand, for the optimization for the reconstruction networks, the corresponding gradients were more reliable, and hence the learning rate was always set to $10^{-3}$. The noise levels in gradients and their reliablity are quantified through the descent rate of the loss while optimizing with a fixed learning rate of $10^{-3}$ at varying batch sizes obtained through gradient accumulation. During gradient accumulation, gradients for the target batch size was obtained by running the network sequentially on multiple single data points repeatedly and accumulating the gradients.

### 2.6. Multi-Resolution

Inspired by SPARKLING [15], the learning of the *k*-space sampling trajectories was performed using a multi-resolution strategy [25] which starts by learning $2^{R^{\max}}$ times decimated sampling trajectories **K** at the maximal $R^{\max}=5$ decimation level. Next, the solution ${\hat{K}}^{2^{R^{\max}}}$ at the resolution level $R^{\max}$ was then interpolated and used as a warm restart for the up-sampled problem at resolution level $R^{\max}-1$.

We used dyadic scaling and trained our trajectory over five decimation levels ($R^{\max}=5$). This implies that the underlying trajectories were optimized first at $2^{5}=32$ decimation level (32 times downsampled trajectory), followed by upscaling the problem by 2, following the decimation levels as 16→8→4→2→1. This multiresolution strategy was instrumental in ensuring fast convergence toward a local minimizer. Indeed, initially the optimization is carried out with faster convergence as we coarsely optimize the *k*-space trajectory over a reduced number of locations ($R=R^{\max}=5$). Then, the process is refined at higher resolutions as we approach convergence (R=1).

### 2.7. Constraints: Projection vs. Penalty

A common method in the literature [18,19,20,33] to enforce these constraints is to add a penalty $L_{\rfloor }\left(K\right)$ to the loss L, which acts like a regularizer on the *k*-space trajectories **K** being optimized. With this, the loss function L becomes:

(8) $$L\left(x,\hat{x},K\right)=L_{r}\left(x,\hat{x}\right)+L_{c}\left(K\right),$$

 where the penalty $L_{c}\left(K\right)$ follows the expression from [19,33]:

(9) $$\begin{array}{c} L_{c}\left(K\right)=\sum_{i=1}^{N_{c}}\sum_{n=1}^{N_{s}}\left(\lambda_{1}\phi_{\alpha }\left(∥{\dot{k}}_{i}{\left[n\right]∥}_{2}\right)+\lambda_{2}\phi_{\beta }{∥{\ddot{k}}_{i}\left[n\right]∥}_{2}\right)+\lambda_{3}\phi_{0}{∥k\left[k_{\mathrm{TE}}\right]∥}_{2} \end{array}$$

with $\phi_{a}\left(x\right)=\max\left(0,x-a\right)$ and $\lambda_{1}$, $\lambda_{2}$ and $\lambda_{3}$ are hyper-parameters to balance the penalty terms with respect to the reconstruction loss $L_{r}$.

However, this penalty-based approach has the following limitations:

- **Need for hyper-parameter tuning:** Under the penalty based formulation, the hyper-parameters $\lambda_{i}$∀i∈{1,2,3} need to be tuned, which requires additional computation. Note that while we can view Equation (8) as an augmented Lagrangian form for the constrained optimization problem Equation (6), the corresponding Karush-Kuhn-Tucker (KKT) conditions are computationally complex to be solved. Further, as we do not satisfy the Slater’s conditions, as the reconstruction loss $L_{r}$ is non-convex, the solutions of the KKT conditions are not guaranteed to be global minima.
- **Influence of gradients and convergence:** With the addition of penalty terms $L_{c}$, the gradient updates involve added gradients from these penalties $\nabla L_{c}$, which influence the overall trajectory development, and hence the final optimized *k*-space trajectories. Gradient updates with these additional gradient terms can no longer guarantee optimal image reconstruction by minimizing the reconstruction loss $L_{r}$.
- **Guarantee of admissibility:** Finally, the optimization of the augmented Lagrangian form does not guarantee that the final optimized *k*-space trajectory **K** satisfies the constraints Equation (1).

To tackle the above issues, we implemented the projector $\Pi_{Q_{\alpha ,\beta }^{N_{c}}}$ from [24] to project the *k*-space trajectories **K** to the feasible set $Q_{\alpha ,\beta }^{N_{c}}$. This results in a projected gradient descent-based optimization of the loss function L, which is given by the following updating step for the *k*-space trajectories **K**:

(10) $$\begin{array}{cc} K^{t+1} & =\Pi_{Q_{\alpha ,\beta }^{N_{c}}}\left(K^{t}-\eta^{t}\nabla_{K}L_{r}\left(x,\hat{x}\right)\right)\;. \end{array}$$

The projected gradient descent formulation gives an equivalent result to optimize the original reconstruction error $L_{r}$, with the indicator function of the feasible set $Q_{\alpha ,\beta }^{N_{c}}$ as the penalty term. However, as the indicator function is non-differentiable, direct use of such a penalty term in auto-differentiation frameworks (as an alternative to the projection step as shown in Equation (10)) generates sub-gradients, which make the optimization process extremely slow due to oscillations as there are multiple sub-gradients at each evaluation point.

Practical implementations involved 50 iterations of the projection algorithm from [24], which was sped up using GPU implementations as shown in [15]. In practice, benchmarking with a very small reconstruction network (NC-PDNet with three iterations, rather than twelve) showed 2.25 s per step for penalty-based schemes, while with projection, the computation time was 3.16 s per step.

### 2.8. Practical Implementations

All our implementations in 2D were carried out on a V100 GPU with 32GB memory, while our 3D implementations needed the next generation A100 GPUs with 80 GB of memory. Most of the memory in 3D was occupied by the activations from the 3D convolutional neural networks used in the image denoising step in NC-PDNet. Memory efficient implementations of NUFFT was carried out by using *tensorflow-nufft* [34], which is based on tensorflow implementations of *cuFINUFFT* [35].

## 3. Results

In this section, we first compare our results with state-of-the-art methods, particularly BJORK [19] and PILOT [18]. Next, we provide an explanation on why our approach outperforms its competitors. In short, the reason is tightly linked to the use of a projection step in the optimization process for enforcing the hardware constraints rather than using penalty terms in the loss function. Finally, we benchmark our jointly learned *k*-space sampling pattern and reconstruction network in 3D by comparing it to SPARKLING trajectories with a learned neural network for image reconstruction.

### 3.1. Comparison with State-of-the-Art Methods in 2D

We learned *k*-space trajectories with $N_{c}=16$ shots and $N_{s}=512$ samples per shot (observation time $T_{\mathrm{obs}}=5.12\;\mathrm{ms}$, raster time Δt=10µs, dwell time δt=2µs). For comparison with an earlier baseline, we used SPARKLING trajectories generated with the learned sampling density using LOUPE [17] as obtained in [16] and trained NC-PDNet [23] as a reconstruction model for it.

**Figure 2:**
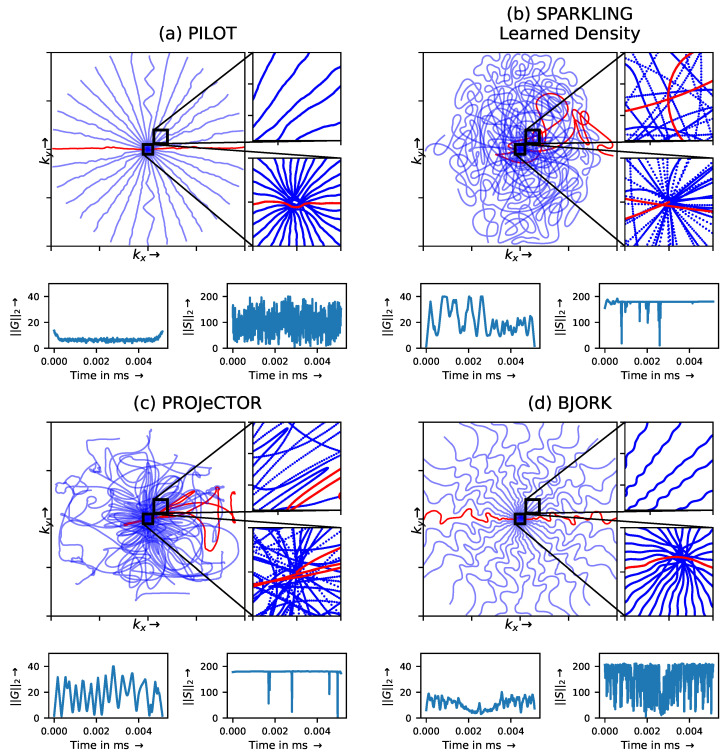
The optimized hardware compliant non-Cartesian *k*-space trajectories using (**a**) PILOT, (**b**) SPARKLING with learned density using LOUPE, (**c**) PROJeCTOR scheme, and (**d**) BJORK. The number of shots is $N_{c}$ = 16. The number of dwell time samples are set to match the same number of sampling points overall. Zoomed in visualizations of the center of *k*-space (bottom) and slightly off-center (top) are presented at the right of the corresponding trajectories. The $\ell_{2}$ norm of the corresponding gradient ${\left||G|\right|}_{2}$ (in mT/m) and slew rate ${\left||S|\right|}_{2}$ (in T/m/s) profiles are depicted below each trajectory.

**Figure 3:**
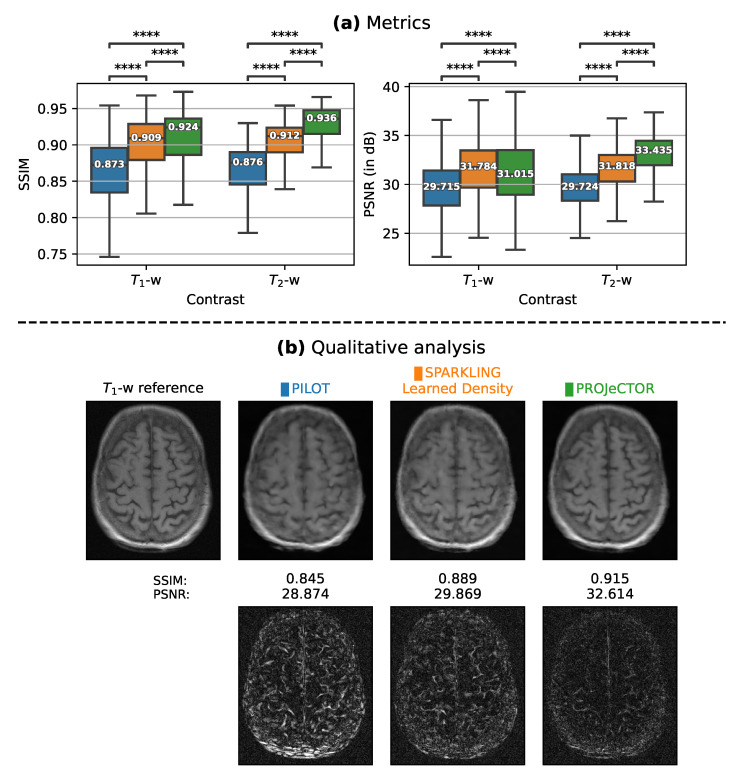
(**a**) Box plots comparing the image reconstruction results on a retrospective study at UF = 2.5 ($N_{c}=16$, $N_{s}=512$, $\frac{\Delta t}{\delta t}=5$) using 512 slices of $T_{1}$ and $T_{2}$ contrasts (fastMRI validation data set) using PILOT (blue), SPARKLING with learned density (orange) and PROJeCTOR (green). SSIMs/PSNRs appear at left/right. The median values of these metrics are highlighted inside the box plots. The significance levels are indicated as a paired *t*-test and are all significant with $p<10^{-4}$ (Significance level indicated with **** between groups in box plots). (**b**) Top: $T_{1}$-w reference image and reconstruction results for a single slice from file_brain_AXT1PRE_209_6001221.h5 with corresponding strategies. (**b**) Bottom: The residuals maps, scaled to match and being comparable across methods.

We compared our results with PILOT and BJORK trajectories (Figure 2), which were obtained directly from the respective authors. As we did not receive their trained reconstruction networks, we trained an NC-PDNet by ourselves for a fair comparison: NC-PDNet makes use of DCp, and its Cartesian version stood second in the 2020 fastMRI challenge [36]. This way, we used the same reconstruction neural network for all the trajectories (with the same parameters), which was trained individually. Our comparison with PILOT (Figure 3) was carried out for $T_{1}$ and $T_{2}$ weighting contrasts in the fastMRI data set.

**Figure 4:**
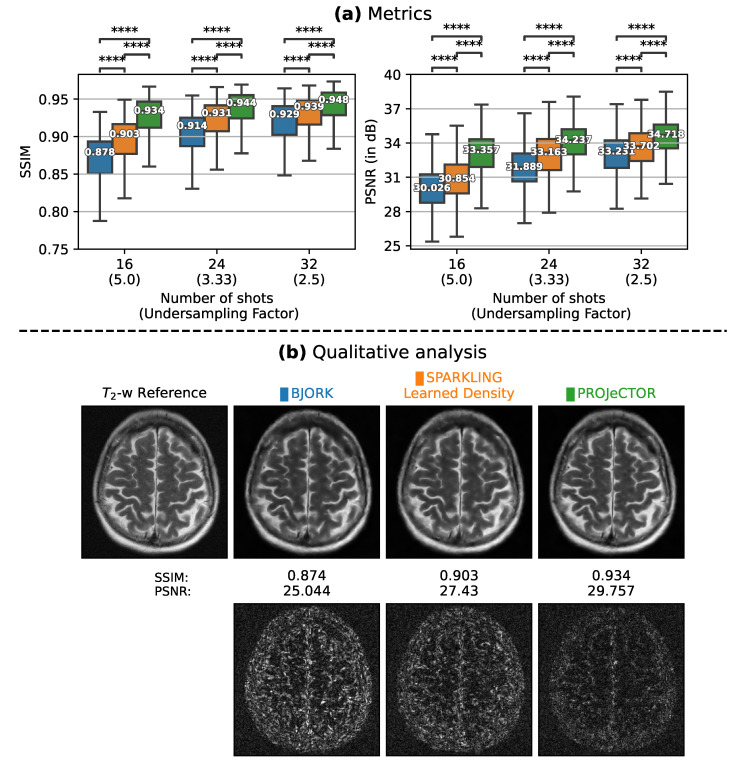
(**a**) Box plots comparing the image reconstruction results on a retrospective study using 512 slices on $T_{2}$ contrast (fastMRI validation dataset) using BJORK (blue), SPARKLING with learned density (orange) and PROJeCTOR (green). The median values of these metrics are highlighted inside the box plots. We present the results at varying UF characterized with $N_{c}=16$, 24 and 32. SSIMs/PSNRs appear at left/right. The significance levels are indicated as paired *t*-test and are all significant with $p<10^{-4}$ (Significance level indicated with **** between groups in box plots). (**b**) Top: $T_{2}$-w reference image and reconstruction results for a single slice from file_brain_AXT2_205_2050175.h5 with corresponding strategies. (**b**) Bottom: The residuals maps, scaled to match and being compared across methods.

As the BJORK trajectory was learned for Δt=4µs, to ensure fair comparison, we obtained trajectories with the same specifications. This comparison (Figure 4) was done at different Undersampling Factor (UF) = $\frac{N^{D}}{N_{c}\times N_{s}}$. Note that UF is a measure of how much the *k*-space is under-sampled with respect to the fully sampled Cartesian *k*-space, while AF reflects on how fast the scan is with respect to the Cartesian reference scan.

We first proceed to analyze the *k*-space trajectories as compared to those yielded by BJORK and PILOT. Then, we compare the reconstruction results of the learned trajectories with BJORK and PILOT.

#### 3.1.1. Trajectory Analysis

When looking at the zoomed portions of optimized trajectories in Figure 2, we observe that PILOT has a hole at the center of *k*-space (cf. the white spot shown in the bottom inset), while BJORK samples the *k*-space densely slightly off the center (cf. bottom inset), which is suboptimal. In contrast, PROJeCTOR and SPARKLING methods sample the central region of *k*-space more densely, which could help obtain improved image quality, notably the contrast.

We also observe at the bottom of each panel in Figure 2 that PILOT and BJORK do not use the hardware gradient capacities at their maximum values and have similar gradient (G(t)) and slew rate (S(t)) profiles, while SPARKLING and PROJeCTOR trajectories are hitting the gradient constraints more often for the maximal gradient and almost everywhere for the slew rate. This difference could be attributed to using a projector for handling hardware constraints in PROJeCTOR and SPARKLING as compared to handling them through penalty terms in PILOT and BJORK.

#### 3.1.2. Retrospective Study

Next, we compared the results of image reconstruction from retrospectively under-sampled *k*-space data using PILOT (Figure 3) and BJORK (Figure 4) trajectories. To this end, we used 512 slices from the fastMRI validation data set. We observe that both SPARKLING with a learned density and PROJeCTOR outperform PILOT and BJORK, with PROJeCTOR yielding the best scores with a gain of nearly 0.06 in SSIM and 3–4 dB in PSNR values as compared to PILOT and BJORK. We computed paired *t*-tests on Structural Similarity Index(SSIM)/Peak Signal-to-Noise Ratio (PSNR) scores between PILOT and PROJeCTOR on one hand and BJORK and PROJeCTOR on the other hand and obtained *p*-values $p<10^{-4}$, thus confirming that the improvements we observed visually and quantitatively are statistically significant.

### 3.2. Hardware Constraints: Penalty vs. Projection

In the above section, we showed how our method outperforms PILOT and BJORK in terms of reconstructed image quality. We assume that these results are due to the different manners that the hardware constraints on the gradients are enforced in the learning process (projector vs. regularizer). To validate this hypothesis, we learned 3D hardware compliant *k*-space sampling trajectories through joint optimization with a reconstruction network using a penalty term instead of a projector.

**Figure 5:**
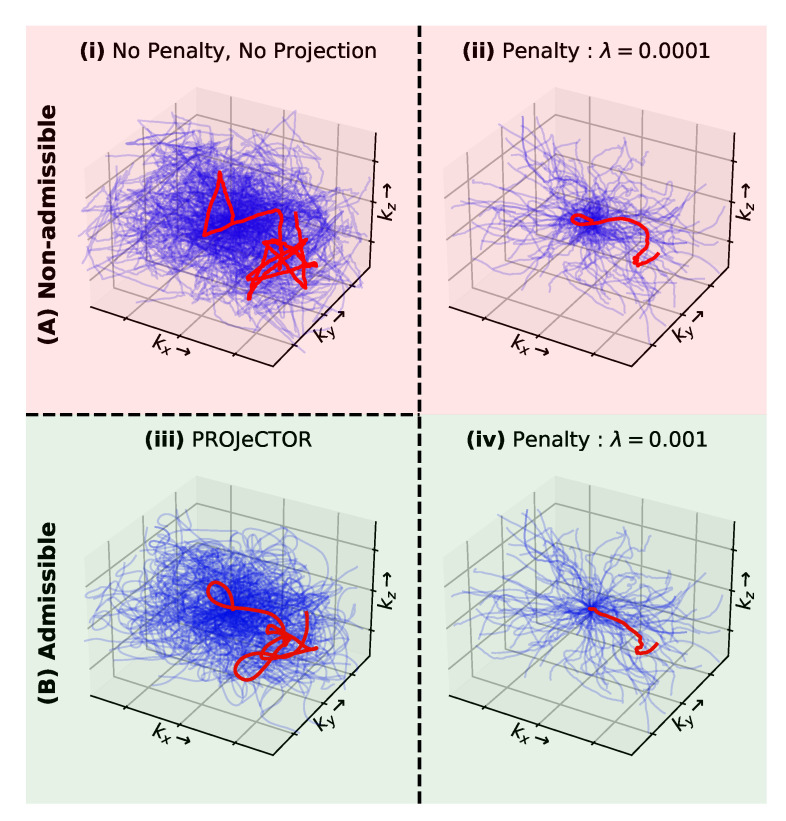
Comparison of (**iii**) PROJeCTOR trajectories with respect to penalty-based versions (**ii**,**iv**). The (**A**) non-admissible trajectories are shaded in red, while (**B**) admissible trajectories are shaded in green. Additionally, unconstrained (no penalty and no projection) trajectories are also presented in (**i**).

**Figure 6:**
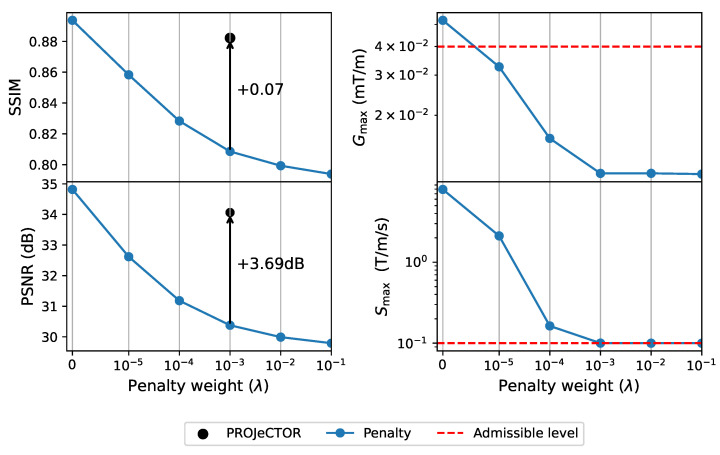
(**Left**): Performance metrics in SSIM and PSNR of penalty-based methods at varying penalty weight λ. The performance PROJeCTOR is also shown for comparison. (**Right**): The feasibility of the penalty-based learned *k*-space trajectories at varying penalty weights (i.e., hyper-parameter) λ, shown by maximum slew rate $S_{\max}$ and maximum gradient strength $G_{\max}$ in the entire sampling pattern. The respective admissible upper levels are drawn with a red-dotted line.

In Figure 5, we present the learned hardware compliant *k*-space sampling trajectories using the projection and penalty-based methods, and then in Figure 6 we depict their corresponding slew rate and gradient profiles. Additionally, we also show in Figure 6 the validation SSIM scores as a function of the penalty weight (λ). For the sake of simplicity, we assume $\lambda =\lambda_{i}$, i∈{1,2,3} and we obtain results for $\lambda =10^{-3}$, which is the lowest level of penalty resulting in hardware-compliant trajectories at the end of training. By doing so, we ensure that we do not influence too much the trajectory shape. However, in our grid search experiments of varying λ across different orders of magnitude, we did not observe any significant drop in validation loss within the range $\left[10^{2},10^{-3}\right]$. Further, to obtain an insightful baseline, we also obtain results for λ=0 corresponding to non-admissible trajectories as we do not enforce any penalty on the gradients and slew rates. Last, we also display the learned trajectories using the PROJeCTOR.

We observed that the best reconstructed image quality can be obtained for λ=0 in terms of validation SSIM and PSNR scores. Further, increasing the weight λ of penalty terms, the validation SSIM and PSNR scores drop as the *k*-space trajectories get more constrained. Interestingly, as $\lambda =10^{-3}$ the *k*-space trajectories are getting hardware compliant (see Figure 5B(iii)), but they become strongly constrained and do not reach the same level of flexibility as those learned by PROJeCTOR. This results in a significant decrease in the performance of penalty-based methods as compared to projection-based methods.

Finally, we observed that using a projection-based method, the *k*-space trajectories are closer to those obtained with λ=0.

### 3.3. Comparison with SPARKLING in 3D

Finally, we compared the performances of our data-driven jointly learned *k*-space trajectories to the model-driven SPARKLING trajectories in 3D imaging. The networks were trained for 240 epochs, with 32 steps per epoch on the Calgary brain data set [27], for trajectories at AF = 20, resulting in $N_{c}=1681$ shots. To ensure a fair comparison, we learned the same NC-PDNet, i.e., image reconstruction neural network for the same number of steps as was done for PROJeCTOR trajectories.

**Figure 7:**
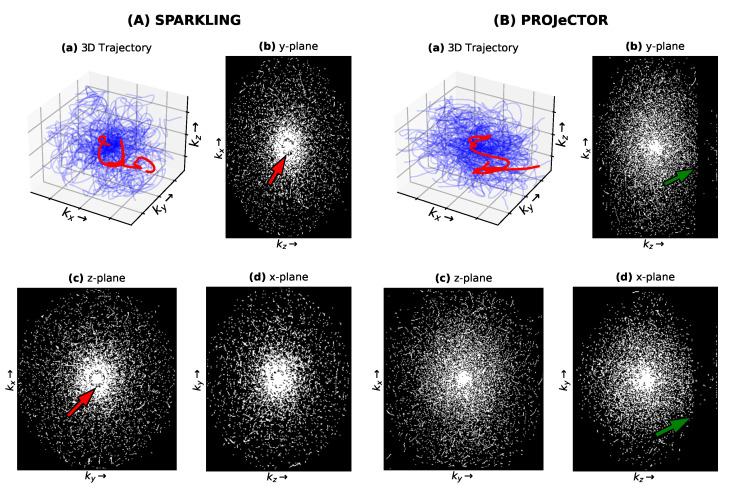
*k*-space sampling trajectories for (**A**) SPARKLING and (**B**) PROJeCTOR. For easier visualization, only 70 shots of 3D trajectory are shown in (**a**). The resulting gridded sampling pattern is shown for mid-plane slices along the (**b**) y-plane, (**c**) z-plane and (**d**) x-plane.

From the mid-slice cuts of gridded sampling patterns in *k*-space in Figure 7A,B(b,d), we see that SPARKLING trajectories present radial-like sampling at the center of *k*-space, which could induce some *k*-space holes (see red arrows in (A.b) and (A.c)). This type of imperfection is not present in the learned PROJeCTOR *k*-space sampling pattern ((B.b) and (B.c)). Further, as the trajectories and reconstruction network were learned on partial Fourier *k*-space data, PROJeCTOR trajectories also learned to exploit this by not sampling these regions (see the dark areas pointed out by green arrows in (B.b) and (B.d)).

**Figure 8:**
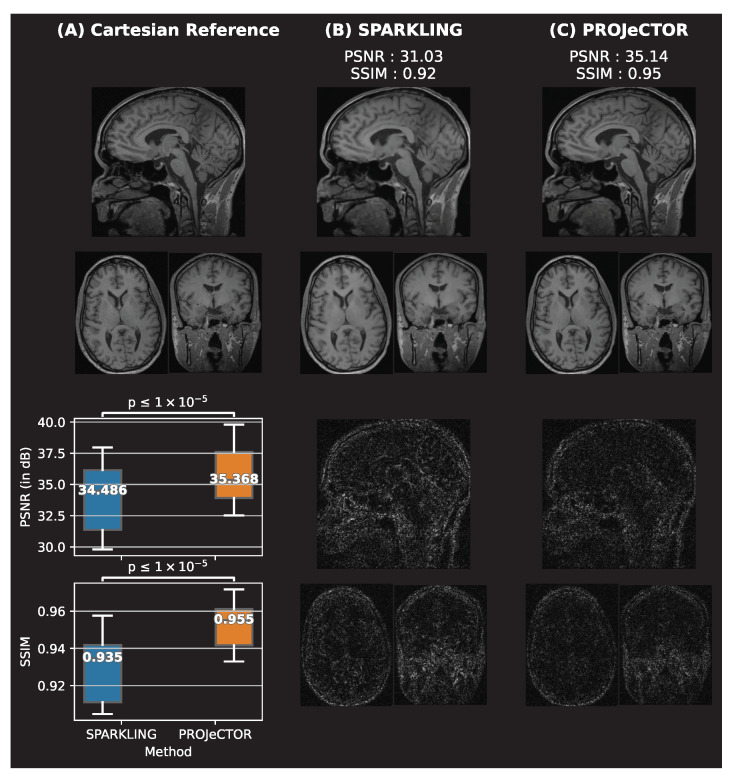
Qualitative and quantitative comparisons of reconstructed images from 3D (**B**) SPARKLING and (**C**) PROJeCTOR trajectories at AF = 20 as compared to (**A**) Cartesian reference. The reconstructed images are shown in the top row, while the residuals are shown in the bottom. Further, box plots of the SSIM and PSNR scores on 20 test data sets are shown in the bottom-left. The significance levels are marked through a paired samples Wilcoxon test.

Finally, comparing the actual reconstructed MR images in Figure 8, we see that the SPARKLING trajectories result in blurrier images, while the PROJeCTOR retains the high-frequency details. This can be observed qualitatively through the residual images and quantitatively through box plots indicating the SSIM and PSNR scores, taken on 20 test data sets. We see that PROJeCTOR outperforms SPARKLING by nearly 0.02 points in SSIM and +2 dB in PSNR scores. As our evaluation is done on 20 matched data points, we use the Wilcoxon signed-rank test, which is a non-parametric statistical hypothesis test used here to compare the locations of two populations using two matched samples. We found that the differences in both the SSIM and PSNR scores are statistically significant with $p<10^{-5}$.

## 4. Discussion

In this work, we present a generic framework for jointly learning the trajectory and image reconstruction neural network. We embedded the projection step from [24] and learned these PROJeCTOR trajectories through a novel projected gradient descent fashion to ensure hardware compliance.

Although the learned neural networks in PILOT [18] and BJORK [19] were not available for a full end-to-end comparison, we performed a fair assessment by training a NC-PDNet [23] as a common deep neural network reference for image reconstruction. Through retrospective studies in 2D on the fastMRI validation data set, we showed that PROJeCTOR works across multiple resolutions and leads to superior performance of the trajectories and improved image quality overall, with a nearly 3–4 dB gain in PSNR value and almost 0.06 gain in SSIM score.

This improvement over state-of-the-art methods can be attributed to the embedded projection step as compared to penalty to ensure hardware compliance. We carried out an ablation study and showed that the projection step is crucial for having significantly improved performance of the learned trajectories, as compared to penalty-based approaches.

Finally, in 3D, we compared the model-driven method SPARKLING with the data-driven method PROJeCTOR and showed a gain of 2 dB in PSNR and 0.02 gain in SSIM in favor of the latter.

Future prospects of this work include prospective implementations through modifications of $T_{1}$ and $T_{2}$-w imaging sequences. Such practical implementations could possibly bring up new sequence-specific constraints on k-space trajectories and also affect the overall performance due to lower Signal-to-Noise Ratio (SNR).

A limitation of current work is that our training paradigm was set-up in an emulated single coil setting as we were limited by memory constraints on GPU. A more realistic implementation would involve a multi-coil imaging setting, which is mandatory to efficiently utilize parallel imaging and become closer to the real data acquisition context, allowing us to reach higher AF. However, this memory bottleneck can be alleviated through efficient transfers between CPU and GPU or multi-GPU implementations. Further, the network can be improved by extending the currently implemented simple forward acquisition model NUFFT to a more realistic and complex model, which takes off-resonance effects due to $B_{0}$ inhomogeneities [37] and gradient imperfections into account. These aspects will be explored in our future works. 

## Data Availability

Not applicable.

## Abbreviations

The following abbreviations are used in this manuscript:

ADC	analog-to-digital converter
AF	Acceleration Factor
BJORK	B-spline parameterized Joint Optimization of Reconstruction and K-space
	trajectories
CS	Compressed Sensing
DCp	Density Compensators
KKT	Karush–Kuhn–Tucker
MRI	Magnetic Resonance Imaging
NUFFT	Non-Uniform Fast Fourier Transform
PILOT	Physics-informed learned optimal trajectories
PROJeCTOR	PROjection for Jointly lEarning non-Cartesian Trajectories while Optimizing
	Reconstructor
PSNR	Peak Signal-to-Noise Ratio
SNR	Signal-to-Noise Ratio
SPARKLING	Spreading Projection Algorithm for Rapid K-space sampLING
SSIM	Structural Similarity Index
TE	Echo Time
TSD	Target Sampling Density
UF	Undersampling Factor
VDS	Variable Density Sampling
